# Labeled RFS-Based Track-Before-Detect for Multiple Maneuvering Targets in the Infrared Focal Plane Array

**DOI:** 10.3390/s151229829

**Published:** 2015-12-08

**Authors:** Miao Li, Jun Li, Yiyu Zhou

**Affiliations:** College of Electronic Science and Engineering, National University of Defense Technology, Changsha 410073, Hunan, P.R. China; junli331@163.com (J.L.); yiyuzyj@126.com (Y.Z.)

**Keywords:** labeled random finite sets, labeled multi-Bernoulli, track-before-detect, maneuvering target, Sequential Monte Carlo

## Abstract

The problem of jointly detecting and tracking multiple targets from the raw observations of an infrared focal plane array is a challenging task, especially for the case with uncertain target dynamics. In this paper a multi-model labeled multi-Bernoulli (MM-LMB) track-before-detect method is proposed within the labeled random finite sets (RFS) framework. The proposed track-before-detect method consists of two parts—MM-LMB filter and MM-LMB smoother. For the MM-LMB filter, original LMB filter is applied to track-before-detect based on target and measurement models, and is integrated with the interacting multiple models (IMM) approach to accommodate the uncertainty of target dynamics. For the MM-LMB smoother, taking advantage of the track labels and posterior model transition probability, the single-model single-target smoother is extended to a multi-model multi-target smoother. A Sequential Monte Carlo approach is also presented to implement the proposed method. Simulation results show the proposed method can effectively achieve tracking continuity for multiple maneuvering targets. In addition, compared with the forward filtering alone, our method is more robust due to its combination of forward filtering and backward smoothing.

## 1. Introduction

The problem of jointly detecting and tracking multiple maneuvering targets in an infrared focal plane array is very challenging and has received great attention in the last several years [1,2,3,4,5,6,7]. In many applications, the estimation is often performed on point measurements after threshold segmentation [8,9,10,11]. However, in situations where the signal-to-noise ratio (SNR) of the infrared sensor is low, threshold segmentation of the sensor output may cause false alarms and missing targets, as the noise level is high enough to generate detections [12,13], so it is necessary to make use of all information in the images to improve the detection and tracking performance. Fortunately, track-before-detect (TBD), or tracking without threshold segmentation is an effective method as shown in [14,15,16]. In addition, in many applications, for example infrared search and track (IRST), precise guidance, and space situation awareness (SSA), the tracking method is required to track all relevant targets which typically exhibit different motion models [6,17,18,19]. This paper investigates the problem of jointly estimating the number of maneuvering targets and their states from infrared image observations.

A recent approach of tracking is to represent the multi-target state as a random finite set (RFS). Based on the RFS framework, the probability hypothesis density (PHD), Cardinalized PHD (CPHD) and multi-target multi-Bernoulli (MeMBer) filters have been proposed [20,21,22,23,24]. The PHD and CPHD recursions propagate the first moment and cardinality distribution of the multi-target random set while the multi-Bernoulli filter propagates the parameters of a multi-Bernoulli distribution that approximates the posterior multi-target density. However, these methods only provide unlabeled point estimates at each time, and additional post-processing is necessary to form tracks. In [25,26], Tuong and Ngu introduced the framework of labeled RFS which augments the state of each target by a track label and the Generalized Labeled Multi-Bernoulli (GLMB) and the δ-GLMB RFS were proposed as the specific subclasses of labeled RFS. The labeled Multi-Bernoulli (LMB) filter proposed in [27], is an efficient approximation of the δ-GLMB filter. For LMB filter, the tracks are supposed to be statistically independent, and the computational cost can be reduced due to partitioning and parallel updates. In [28,29], GLMB TBD and LMB TBD were applied in visual tracking and radar tracking, respectively. In our work, we will extend the LMB TBD filter with a special focus on point target tracking in the infrared focal plane array.

A single-model LMB filter may fail to track maneuvering targets whose motion model may switch between different models, because the motion model of the filter does not match the actual dynamics. It is well known that the Interacting Multiple Models (IMM) approach has been proven to be very effective and has been adopted in many applications to deal with this challenge [30]. Stephan *et al*. adopted the IMM approach based on LMB filter to tackle maneuvering targets [31]. However, it is only applicable to point measurements after threshold segmentation.

In addition, target intensity may change due to projection location, imaging distance, optic angle of sensor, atmosphere environments, and so on, so in this case, the estimation performance is limited. A delayed decision which incorporates multiple image frames can improve estimation performance, because multiple image frames can provide more information. Recently, smoothing has been adopted within the Bernoulli RFS framework. [12,32] introduced the Bernoulli backward smoothers for point measurements and image observations, respectively. However, they can only smooth single-model single targets.

To solve the aforementioned problems, we propose a novel multiple maneuvering target track-before-detect method within the labeled RFS framework, referred to as multi-model labeled multi-Bernoulli (MM-LMB) TBD method. The MM-LMB TBD method consists of an MM-LMB filter and an MM-LMB smoother. The MM-LMB filter propagates multi-target density in the forward direction, and the MM-LMB smoother optimizes the history multi-target density with current data. Firstly, the original LMB filter is extended to a LMB filter for TBD based on the target and measurement models in the infrared focal plane array. Secondly, by integrating the IMM approach with the LMB filter, a MM-LMB filter for TBD is derived. Thirdly, taking advantage of the track labels provided by labeled RFS and posterior model transition probability, the MM-LMB smoother for TBD is presented, which extends single-model single-target smoothing to multi-model multi-target smoothing. This work also provides an efficient implementation based on particle or Sequential Monte Carlo (SMC) approximation [33,34], and demonstrates significantly improved detection and tracking performance in a typical multiple maneuvering target scenario.

The remainder of the paper is organized as follows: Section 2 introduces the notation used in this paper and reviews the theories of labeled RFS and labeled multi-Bernoulli RFS. Section 3 proposes the MM-LMB TBD method and presents the derivation of the MM-LMB filter and MM-LMB smoother for TBD in detail. Sequential Monte Carlo implementation is also discussed in Section 3. The results and analysis of the experiments are mainly presented in Section 4. Section 5 draws the conclusions.

## 2. Background

This section introduces the notation and provides a brief review of labeled RFS and the labeled multi-Bernoulli RFS. For more details, the reader is referred to [27,35].

### 2.1. Notation

In this paper, small letters (e.g., *x*) are used to denote single-target states and capital letters (e.g., *X*) are used to denote multi-target states. Labeled target states are indicated by boldface letters (e.g., ***x***, ***X***). In addition, spaces are represented by blackboard bold letters (e.g., X denotes the state space). Image observation at time k is denoted by $z_{k}$. The inner product is denoted by 〈f,g〉≜∫f(x)g(x)dx. The multi-target exponential of a real valued function h raised to a set X is defined as $h^{X}≜\prod_{x\in X}h\left(x\right)$, where $h^{\phi }=1$ by convention. The generalized Kronecker delta function and inclusion function are denoted by $\delta_{Y}\left(\cdot \right)$ and $1_{Y}\left(\cdot \right)$, respectively.

### 2.2. Labeled RFS

In order to jointly estimate the targets’ states and their individual tracks, labeled RFS was introduced based on the RFS framework [25]. A label $\ell \in L=\left\{\alpha_{i}:i\in \mathbb{N}\right\}$ is appended to the state x∈X to enable the estimation of a target track. L is a discrete space, whose elements are distinct and the space ℕ denotes the set of positive integers. Hence, a labeled single-target state and a labeled multi-target state can be described by x=(x,ℓ) and $X=\left\{x^{\left(1\right)},\cdots ,x^{\left(n\right)}\right\}$, respectively.

Let ℒ:X×L→L be the projection ℒ((x,ℓ))=ℓ, the set of track labels of the labeled RFS X is obtained by ℒ(X)={ℒ(x):x∈X}. The labels are required to be distinct. It means that the cardinalities of the set of labels and the set of state vectors are identical. It is mathematically ensured using the distinct label indicator $\Delta \left(X\right)=\delta_{\left|X\right|}\left(\left|ℒ\left(X\right)\right|\right)$.

### 2.3. Labeled Multi-Bernoulli RFS

The single target density can be modeled by Bernoulli RFS. Conditional on $X_{k-1}=\emptyset$, the target can re-enter or appear with probability $p_{b,k|k-1}$ and occupy kinematic state $x_{k}$ with probability density $b_{k|k-1}\left(x_{k}\right)$, or remain absent or disappeared with probability $1-p_{b,k|k-1}$. It can be described by the Bernoulli RFS as following [32]:

(1) $$p_{k|k-1}\left(X_{k}|\emptyset \right)=\{\begin{array}{l} 1-p_{b,k|k-1},\\ p_{b,k|k-1}b_{k|k-1}\left(x_{k}\right), \end{array}\begin{array}{l} X_{k}=\emptyset \\ X_{k}=\left\{x_{k}\right\} \end{array}$$

In addition, conditional on $X_{k-1}=\left\{x_{k-1}\right\}$ , the target can survive and acquire a new state $x_{k}$ with probability density $p_{S,k|k-1}\left(x_{k-1}\right)f_{k|k-1}\left(x_{k}|x_{k-1}\right)$, or disappear with probability $1-p_{S,k|k-1}\left(x_{k-1}\right)$. It can be described by the following Bernoulli RFS [12]:

(2) $$p_{k|k-1}\left(X_{k}|\left\{x_{k|k-1}\right\}\right)=\{\begin{array}{l} 1-p_{S,k|k-1}\left(x_{k-1}\right),\\ p_{S,k|k-1}\left(x_{k-1}\right)f_{k|k-1}\left(x_{k}|x_{k-1}\right), \end{array}\begin{array}{l} X_{k}=\emptyset \\ X_{k}=\left\{x_{k}\right\} \end{array}$$

A multi-Bernoulli RFS X can be regarded as a union of independent Bernoulli RFSs $X^{\left(i\right)}$ with existence probability $r^{\left(i\right)}$ and probability density $p^{\left(i\right)}$, *i.e*., $X=\cup_{i=1}^{M}X^{\left(i\right)}$. Then, parameter set ${\left\{r^{\left(i\right)},p^{\left(i\right)}\right\}}_{i=1}^{M}$ is used to represent a multi-Bernoulli RFS.

Like a multi-Bernoulli RFS, a labeled multi-Bernoulli RFS with state space X and label space L can be described by the parameter set $\pi ={\left\{r^{\left(\ell \right)},p^{\left(\ell \right)}\right\}}_{\ell \in L}$. The components ℓ are assumed to be statistically independent [27,35]. A label ℓ=(k,i) is assigned to each target which is a pair of the time of birth k and a label index i∈ℕ. Then, the label space for new targets born at time k is denoted as $L_{k}$, and the new target born at time k has state $x\in X\times L_{k}$. The label space for all targets at time k can be denoted as $L_{0:k}$, which is constructed recursively by $L_{0:k}=L_{0:k-1}\cup L_{k}$. In addition, the LMB RFS can also be represented in the form of GLMB RFS:

(3) $$\pi \left(X\right)=\Delta \left(X\right)\omega \left(ℒ\left(X\right)\right)p^{X}$$

where the weights and the spatial distributions are given as follows [27]:

(4) $$\omega \left(L\right)=\prod_{i\in L}\left(1-r^{\left(i\right)}\right)\prod_{\ell \in L}\frac{\left(1_{L}\left(\ell \right)r^{\left(\ell \right)}\right)}{1-r^{\left(\ell \right)}}$$

(5) $$p\left(x,\ell \right)=p^{\left(\ell \right)}\left(x\right)$$

## 3. The Multi-Model LMB TBD

### 3.1. Target and Measurement Models

In this paper, the observation is a two-dimensional image generated by infrared focal plane array. The pixel i of image can be indexed by the row and column coordinates, *i.e*., i=(a,b). The image is regarded as being made up of the sum of target signals and sensor noise signals. For long-range surveillance applications, the target is close to a point source, referred to as point target [36]. Thus, the sampled target spatial signature can be well modeled by the following 2D Gaussian shape [12,37]:

(6) $$h_{i}\left(x\right)=\frac{\Delta_{a}\Delta_{b}I}{2\pi \Sigma^{2}}\exp\left(-\frac{{\left(\Delta_{a}a-x_{a}\right)}^{2}+{\left(\Delta_{b}b-x_{b}\right)}^{2}}{2\Sigma^{2}}\right)$$

where Σ is blurring factor, $\left(x_{a},x_{b}\right)$ is the position of the state x, I is the target intensity. Each cell corresponds to a rectangular region of dimensions $\Delta_{a}\times \Delta_{b}$. $h_{i}\left(x\right)$ is the contribution to pixel i from the state x, which depends on the blurring factor, target position and target intensity.

A target with state x illuminates a set of pixels denoted by T(x). The T(x) is regarded as the square region whose center is closest to the position of x. Let the measurement likelihood in pixel i in the presence of a target with state x be denoted by $\phi^{\left(i\right)}\left(z^{\left(i\right)},x\right)$, and the likelihood under the hypothesis of no targets be $\varphi^{\left(i\right)}\left(z^{\left(i\right)}\right)$, as Equation (7). More details on the measurement likelihood can be found in [38]:

(7) $$p\left(z^{\left(i\right)}|x\right)=\{\begin{array}{l} \varphi \left(z^{\left(i\right)},x\right),\\ \phi \left(z^{\left(i\right)}\right), \end{array}\begin{array}{c} i\in T\left(x\right)\\ i\notin T\left(x\right) \end{array}$$

Then, the point target model is incorporated into the likelihood function. The measurement likelihood is given as follows:

(8) $$\phi^{\left(i\right)}\left(z^{\left(i\right)}\right)=N\left(z^{\left(i\right)};0,\sigma \right)$$

(9) $$\varphi^{\left(i\right)}\left(z^{\left(i\right)},x\right)=N\left(z^{\left(i\right)};h_{i}\left(x\right),\sigma \right)$$

Conditioned on the multi-target state X, the multi-target likelihood of z is the product over all cells [38], *i.e*.:

(10) $$g\left(z|X\right)=f\left(z\right)\prod_{x\in X}\gamma_{z}\left(x\right)$$

where:

(11) $$f\left(z\right)=\prod_{i=1}^{m}\varphi^{\left(i\right)}\left(z^{\left(i\right)}\right)$$

(12) $$\gamma_{z}\left(x\right)=\prod_{i\in T\left(x\right)}\frac{\phi^{\left(i\right)}\left(z^{\left(i\right)},x\right)}{\varphi^{\left(i\right)}\left(z^{\left(i\right)}\right)}$$

Note that the targets are not closely space targets in this paper. We assume they are not very close to each other, so the separable likelihood model is reasonable in this application.

### 3.2. Multi-Model LMB Filter for TBD

In our method, MM-LMB TBD consists of the MM-LMB filter and MM-LMB smoother for TBD. MM-LMB filter means that the target density is propagated forward from time k to time l>k. MM-LMB smoother means that the target density is propagated backward from time l to time k. This subsection gives the derivation of the forward filter. To derive the MM-LMB filter for TBD, the key issue is to construct the LMB parameter set. For multi-model filter, augmented state (x,τ) is typically used to represent the target’s state and the according motion model τ∈T. $T=\left\{1,2,\cdots ,M_{\tau }\right\}$ denotes the discrete space of all possible motion models. In the context of labeled RFS, the state vector is further augmented by the track label ℓ, as (x,ℓ,τ). Thus, multi-target state is consequently given by:

(13) $$X=\left\{x_{1},\cdots ,x_{n}\right\}=\left\{\left(x_{1},\ell_{1},\tau_{1}\right),\cdots ,\left(x_{n},\ell_{n},\tau_{n}\right)\right\}$$

Let $h_{st}=p_{k|k-1}\left\{\tau_{k}=t|\tau_{k-1}=s\right\}$ denotes the probability that a track switches from model s to model t, where s,t∈T. $H=\left[h_{st}\right]$ denotes the model transition probability matrix, and $\sum_{t=1}^{M_{\tau }}h_{st}=1$.

Each track is represented by its existence probability $r^{\left(\ell \right)}$ and its joint spatial distribution $p^{\left(\ell \right)}\left(x,\tau \right)=p^{\left(\ell \right)}\left(x|\tau \right)p^{\left(\ell \right)}\left(\tau \right)$. $p^{\left(\ell \right)}\left(\tau \right)$ denotes the probability of track ℓ with model τ and $p^{\left(\ell \right)}\left(x|\tau \right)$ denotes the spatial distribution of track ℓ conditioned on model τ.

In order to tackle the problem of maneuvering target TBD, IMM approach is integrated with the LMB filter as MM-LMB filter. Thus, in addition to the prediction stage and update stage, mixing stage is introduced. The recursion of MM-LMB filter for TBD is given as follows:

*Step 1.* *Mixing*: If at time k−1 the posterior density is assumed to be an LMB RFS on the augmented space, which is given by the parameter set as:

(14) $$\pi_{k-1}={\left\{\left(r_{k-1}^{\left(\ell \right)},p_{k-1}^{\left(\ell \right)}\left(x_{k-1}^{\left(\ell \right)},\tau_{k-1}^{\left(\ell \right)}\right)\right)\right\}}_{\ell \in L_{k-1}}$$

Then, the parameter set after mixing can be expressed by:

(15) $${\tilde{\pi }}_{k-1}={\left\{\left(r_{k-1}^{\left(\ell \right)},{\tilde{p}}_{k-1}^{\left(\ell \right)}\left(x_{k-1}^{\left(\ell \right)},\tau_{k}^{\left(\ell \right)}\right)\right)\right\}}_{\ell \in L_{k-1}}$$

For the IMM approach, the motion model transition is assumed to be independent of the target’s state transition. It is only decided by motion model transition probability. Thus, ${\tilde{p}}_{k-1}^{\left(\ell \right)}\left(x_{k-1}^{\left(\ell \right)},\tau_{k}^{\left(\ell \right)}\right)$ can be calculated by:

(16) $$\begin{array}{cc} {\tilde{p}}_{k-1}^{\left(\ell \right)}\left(x_{k-1}^{\left(\ell \right)},\tau_{k}^{\left(\ell \right)}=t\right) & =\sum_{s=1}^{M_{\tau }}p_{k-1}^{\left(\ell \right)}\left(x_{k-1}^{\left(\ell \right)},\tau_{k}^{\left(\ell \right)}=t,\tau_{k-1}^{\left(\ell \right)}=s\right)\\ & =\sum_{s=1}^{M_{\tau }}p_{k-1}^{\left(\ell \right)}\left(\tau_{k}^{\left(\ell \right)}=t|x_{k-1}^{\left(\ell \right)},\tau_{k-1}^{\left(\ell \right)}=s\right)p_{k-1}^{\left(\ell \right)}\left(x_{k-1}^{\left(\ell \right)},\tau_{k-1}^{\left(\ell \right)}=s\right)\\ & =\sum_{s=1}^{M_{\tau }}h_{st}p_{k-1}^{\left(\ell \right)}\left(x_{k-1}^{\left(\ell \right)},\tau_{k-1}^{\left(\ell \right)}=s\right) \end{array}$$

*Step 2.* *Prediction*: Then, the predicted LMB RFS of the multi-model LMB filter is given by the parameter set:

(17) $$\pi_{k|k-1}={\left\{\left(r_{P,k|k-1}^{\left(\ell \right)},p_{P,k|k-1}^{\left(\ell \right)}\left(x_{k|k-1},\tau_{k}\right)\right)\right\}}_{\ell \in L_{k-1}}\cup {\left\{\left(r_{B}^{\left(\ell \right)},p_{B}^{\left(\ell \right)}\left(x_{k},\tau_{k}\right)\right)\right\}}_{\ell \in B}$$

where:

(18) $$r_{P,k|k-1}^{\left(\ell \right)}=r_{k-1}^{\left(\ell \right)}\eta \left(\ell \right)$$

(19) $$p_{P,k|k-1}^{\left(\ell \right)}\left(x,\tau \right)=\frac{\langle p_{S}^{\left(\ell \right)}\left(\cdot ,\tau \right)f^{\left(\ell \right)}\left(x|\cdot ,\tau \right),{\tilde{p}}_{k-1}^{\left(\ell \right)}\left(\cdot ,\tau \right)\rangle }{\eta \left(\ell \right)}$$

(20) $$\begin{array}{cc} \eta \left(\ell \right) & =\sum_{\tau \in T}\int p_{S}^{\left(\ell ,\tau \right)}\left(x,\tau \right){\tilde{p}}_{k-1}^{\left(\ell \right)}\left(x,\tau \right)dx\\ & =\sum_{\tau \in T}{\tilde{p}}_{k-1}^{\left(\ell \right)}\left(\tau \right)\int p_{S}^{\left(\ell ,\tau \right)}\left(x,\tau \right){\tilde{p}}_{k-1}^{\left(\ell \right)}\left(x|\tau \right)dx \end{array}$$

The terms $p_{S}^{\left(\ell \right)}\left(\cdot \right)$ and $f^{\left(\ell \right)}\left(x|\cdot ,\tau \right)$ denote the survival probability and single-target transition density of track ℓ at model τ, respectively. In most applications, the survival probability of the tracks can be assumed to be independent of the current motion model, *i.e*., $p_{S}^{\left(\ell \right)}\left(x,\tau \right)=p_{S}^{\left(\ell \right)}\left(x\right)$. ${\tilde{p}}_{k-1}^{\left(\ell \right)}\left(\tau \right)$ and ${\tilde{p}}_{k-1}^{\left(\ell \right)}\left(x|\tau \right)$ denote the model probability and conditional spatial distribution of track ℓ after mixing at time k−1. As shown in Equation (17), the LMB parameter set for the predicted multi-target density $\pi_{k|k-1}$ is formed by the union of LMB parameter sets for the survival targets and birth targets. The first term in Equation (17) represents survival targets and the second one denotes birth targets. B is the label space of birth targets, and $L_{k|k-1}=L_{k-1}\cup B$.

*Step 3.* *Update from image observation*: We rewrite the predicted multi-target density as a typical LMB RFS, which is given by the parameter set:

(21) $$\pi_{k|k-1}={\left\{\left(r_{k|k-1}^{\left(\ell \right)},p_{k|k-1}^{\left(\ell \right)}\left(x,\tau \right)\right)\right\}}_{\ell \in L_{k|k-1}}$$

Reference [29] derived the update procedure for single-model TBD. For the case with multiple motion models, the existence probability $r_{k}^{\left(\ell \right)}$ and spatial density $p_{k}^{\left(\ell \right)}\left(x,\tau \right)$ can be updated by Equations (22) and (23), respectively:

(22) $$r_{k}^{\left(\ell \right)}=\frac{r_{k|k-1}^{\left(\ell \right)}\eta_{z}\left(\ell \right)}{1-r_{k|k-1}^{\left(\ell \right)}+r_{k|k-1}^{\left(\ell \right)}\eta_{z}\left(\ell \right)}$$

(23) $$p_{k}^{\left(\ell \right)}\left(x,\tau \right)=\frac{p_{k|k-1}^{\left(\ell \right)}\left(x,\tau \right)\gamma_{z}\left(x,\ell \right)}{\eta_{z}\left(\ell \right)}$$

where $\gamma_{z}\left(\cdot \right)$ has been given in Section 3.1, and:

(24) $$\eta_{z}\left(\ell \right)=\sum_{\tau \in T}\eta_{z}\left(\ell ,\tau \right)$$

(25) $$\begin{array}{cc} \eta_{z}\left(\ell ,\tau \right) & =\int \gamma_{z}\left(x,\ell \right)p_{k|k-1}^{\left(\ell \right)}\left(x,\tau \right)dx\\ & =p_{k|k-1}^{\left(\ell \right)}\left(\tau \right)\int \gamma_{z}\left(x,\ell \right)p_{k|k-1}^{\left(\ell \right)}\left(x|\tau \right)dx \end{array},$$

where $p_{k|k-1}^{\left(\ell \right)}\left(\tau \right)$ and $p_{k|k-1}^{\left(\ell \right)}\left(x|\tau \right)$ represent the model probability and conditional spatial distribution of track ℓ with model τ. In fact, the Equation (22) can be interpreted as the union of two cases: target appears and target disappears. $r_{k|k-1}^{\left(\ell \right)}\eta_{z}\left(\ell \right)$ denotes the probability when target appears. $1-r_{k|k-1}^{\left(\ell \right)}$ denotes the probability when target disappears.

### 3.3. Multi-Model LMB Smoother for TBD

In the backward smoothing, the smoothed target density is propagated backward from time l to time k<l. In essence, we want to optimize the history target density by the measurement data up to time l>k. Taking advantage of the target labels, the multi-target backward smoothing can be realized with an approach similar to that of single-target backward smoothing. Multiple targets are independent, and can be smoothed respectively. More details on single-target backward smoothing can be found in [12]. The recursion is initialized with l=k. The smoothed LMB density of track ℓ from time l to k is denoted by $\pi_{k|l}={\left\{\left(r_{k|l}^{\left(\ell \right)},p_{k|l}^{\left(\ell \right)}\right)\right\}}_{\ell \in L}$. Then the smoothed LMB density $\pi_{k-1|l}={\left\{\left(r_{k-1|l}^{\left(\ell \right)},p_{k-1|l}^{\left(\ell \right)}\right)\right\}}_{\ell \in L}$ can be calculated by:

(26) $$r_{k-1|l}^{\left(\ell \right)}=1-\left(1-r_{k-1|k-1}^{\left(\ell \right)}\right)\left(\alpha_{B,k|l}^{\left(\ell \right)}+\beta_{B,k|l}^{\left(\ell \right)}\sum_{\tau^{\prime }\in T}\int \frac{p_{k|l}^{\left(\ell \right)}\left(x^{\prime },\tau^{\prime }\right)}{p_{k|k-1}^{\left(\ell \right)}\left(x^{\prime },\tau^{\prime }\right)}b_{k|k-1}^{\left(\ell \right)}\left(x^{\prime },\tau^{\prime }\right)dx^{\prime }\right)$$

(27) $$p_{k-1|l}^{\left(\ell \right)}\left(x,\tau \right)\propto p_{k-1|k-1}^{\left(\ell \right)}\left(x,\tau \right)\left(\alpha_{S,k|l}^{\left(\ell \right)}+\beta_{S,k|l}^{\left(\ell \right)}\left(x,\tau \right)\sum_{\tau^{\prime }\in T}\int \frac{p_{k|l}^{\left(\ell \right)}\left(x^{\prime },\tau^{\prime }\right)}{p_{k|k-1}^{\left(\ell \right)}\left(x^{\prime },\tau^{\prime }\right)}f_{k|k-1}^{\left(\ell \right)}\left(x^{\prime },\tau^{\prime }|x,\tau \right)dx^{\prime }\right)$$

where:

(28) $$\alpha_{B,k|l}^{\left(\ell \right)}=\left(1-p_{b}\right)\frac{\left(1-r_{k|l}^{\left(\ell \right)}\right)}{\left(1-r_{k|k-1}^{\left(\ell \right)}\right)}$$

(29) $$\beta_{B,k|l}^{\left(\ell \right)}=p_{b}\frac{r_{k|l}^{\left(\ell \right)}}{r_{k|k-1}^{\left(\ell \right)}}$$

(30) $$\alpha_{S,k|l}^{\left(\ell \right)}\left(x,\tau \right)=\left(1-p_{S,k|k-1}^{\left(\ell \right)}\left(x,\tau \right)\right)\cdot \frac{\left(1-r_{k|l}^{\left(\ell \right)}\right)}{\left(1-r_{k|k-1}^{\left(\ell \right)}\right)}$$

(31) $$\beta_{S,k|l}^{\left(\ell \right)}\left(x,\tau \right)=p_{S,k|k-1}^{\left(\ell \right)}\left(x,\tau \right)\cdot \frac{r_{k|l}^{\left(\ell \right)}}{r_{k|k-1}^{\left(\ell \right)}}$$

$\left(x^{\prime },\tau^{\prime }\right)$ is the augmented states of smoothing from l to k, $f\left(x^{\prime },\tau^{\prime }|x,\tau \right)$ is single target transition density at time k, given target state x and motion model τ. The model transition is independent of the state transition, *i.e*., $f\left(x^{\prime },\tau^{\prime }|x,\tau \right)=h_{\tau \tau^{\prime }}f\left(x^{\prime }|x,\tau \right)$.

Like the analysis above, the motion model transition probability has an important influence on target state estimation. Backward smoothing makes it possible to get more accurate posterior model transition probability. The posterior model transition probability of track ℓ from k−1 to k, *i.e*., ${\tilde{h}}_{\tau_{k-1}\tau_{k}}^{\left(\ell \right)}$, is calculated as Equation (32). The posterior model transition probability, rather than the prior one, is used to calculate smoothed target density:

(32) $${\tilde{h}}_{\tau_{k-1}\tau_{k}}^{\left(\ell \right)}=p_{k|k}^{\left(\ell \right)}\left(\tau_{k}|\tau_{k-1}\right)=\frac{\int p_{k|k}^{\left(\ell \right)}\left(x,\tau_{k},\tau_{k-1}\right)dx}{\sum_{\tau_{k}\in T}\int p_{k|k}^{\left(\ell \right)}\left(x,\tau_{k},\tau_{k-1}\right)dx}$$

### 3.4. Sequential Monte Carlo Implementation

In this subsection, we use the SMC approach to implement the MM-LMB TBD. The single target density at time k−1 is given by a labeled Bernoulli RFS with existence probability $r_{k-1}^{\left(\ell \right)}$ and spatial density $p_{k-1}^{\left(\ell \right)}\left(x_{k-1}^{\left(\ell \right)},\tau_{k-1}^{\left(\ell \right)}\right)$. The spatial density is approximated using a set of weighted particles ${\left\{x_{k-1}^{\left(\ell ,j\right)},\tau_{k-1}^{\left(\ell ,j\right)},\omega_{k-1}^{\left(\ell ,j\right)}\right\}}_{j=1}^{J_{k-1}^{\left(\ell \right)}}$, *i.e*., $p_{k-1}^{\left(\ell \right)}\left(x,\tau \right)=\sum_{j=1}^{J_{k-1}^{\left(\ell \right)}}\omega_{k-1}^{\left(\ell ,j\right)}\delta_{x_{k-1}^{\left(\ell ,j\right)},\tau_{k-1}^{\left(\ell ,j\right)}}\left(x,\tau \right)$. $J_{k-1}^{\left(\ell \right)}$ is the number of particles. Notice that the particles consist of the state and model information with associated weights.

*Mixing and Prediction*: The model samplings for survival targets and birth targets are performed based on the proposals $\alpha_{k}\left(\cdot \right)$ and $\beta_{k}\left(\cdot \right)$. Weights are calculated based on the model transition probability and birth model, respectively. Thus, the model values and corresponding weights can be obtained by:

(33) $$\tau_{k}^{\left(\ell ,j\right)}∼\{\begin{array}{l} \alpha_{k}\left(\cdot |\tau_{k-1}^{\left(\ell ,j\right)}\right),\\ \beta_{k}\left(\cdot \right)\;, \end{array}\begin{array}{l} \ell \in L_{k-1},j=1:J_{k-1}^{\left(\ell \right)}\\ \ell \in B,j=1:J_{B}^{\left(\ell \right)} \end{array}$$

(34) $${\omega^{\prime }}_{k|k-1}^{\left(\ell ,j\right)}=\left\{ \begin{array}{ll} \frac{h_{k|k-1}\left(\tau_{k}^{\left(\ell ,j\right)}|\tau_{k-1}^{\left(\ell ,j\right)}\right)\omega_{k-1}^{\left(\ell ,j\right)}}{\alpha_{k}\left(\tau_{k}^{\left(\ell ,j\right)}|\tau_{k-1}^{\left(\ell ,j\right)}\right)}, & \ell \in L_{k-1},j=1:J_{k-1}^{\left(\ell \right)}\\ \frac{\theta_{k}\left(\tau_{k}^{\left(\ell ,j\right)}\right)}{\beta_{k}\left(\tau_{k}^{\left(\ell ,j\right)}\right)J_{B}^{\left(\ell \right)}}, & \ell \in B,j=1:J_{B}^{\left(\ell \right)} \end{array}\right.$$

where, $h_{k|k-1}\left(\cdot \right)$ and $\theta_{k}\left(\cdot \right)$ are the model transition probability of survival targets and the model distribution of birth targets. $J_{B}$ denotes the number of particles of the birth target. Note that the predicted motion models are sampled from discrete space T.

Then, the particles $x_{k|k-1}^{\left(\ell ,j\right)}$ and weights $\omega_{k|k-1}^{\left(\ell ,j\right)}$ can be generated as follows. The particles and weights for survival targets are drawn conditioned on model $\tau_{k}$, and those for birth targets are obtained based on birth model:

(35) $$x_{k|k-1}^{\left(\ell ,j\right)}∼\{\begin{array}{l} q_{k}\left(\cdot |x_{k-1}^{\left(\ell ,j\right)},\tau_{k}^{\left(\ell ,j\right)}\right),\\ s_{k}\left(\cdot \right)\;, \end{array}\begin{array}{l} \ell \in L_{k-1},j=1:J_{k-1}^{\left(\ell \right)}\\ \ell \in B,j=1:J_{B}^{\left(\ell \right)} \end{array}$$

(36) $$\omega_{k|k-1}^{\left(\ell ,j\right)}=\left\{ \begin{array}{ll} \frac{p_{S,k|k-1}\left(x_{k-1}^{\left(\ell ,j\right)},\tau_{k}^{\left(\ell ,j\right)}\right)f_{k|k-1}\left(x_{k|k-1}^{\left(\ell ,j\right)}|x_{k-1}^{\left(\ell ,j\right)},\tau_{k}^{\left(\ell ,j\right)}\right){\omega^{\prime }}_{k-1}^{\left(\ell ,j\right)}}{q_{k}\left(x_{k}^{\left(\ell ,j\right)}|x_{k-1}^{\left(\ell ,j\right)},\tau_{k}^{\left(\ell ,j\right)}\right)}, & \ell \in L_{k-1},j=1:J_{k-1}^{\left(\ell \right)}\\ \frac{b_{k|k-1}\left(x_{k}^{\left(\ell ,j\right)}|\tau_{k}^{\left(\ell ,j\right)}\right){\omega^{\prime }}_{k-1}^{\left(\ell ,j\right)}}{s_{k}\left(x_{k}^{\left(\ell ,j\right)}|\tau_{k}^{\left(\ell ,j\right)}\right)}, & \ell \in B,j=1:J_{B}^{\left(\ell \right)} \end{array}\right.$$

where, $q_{k}\left(\cdot \right)$ and $s_{k}\left(\cdot \right)$ are the proposals for the survival target and the birth target.

*Update*: Suppose the predicted density is given as $\pi_{k|k-1}={\left\{r_{k|k-1}^{\left(\ell \right)},p_{k|k-1}^{\left(\ell \right)}\left(x_{k|k-1}^{\left(\ell \right)},\tau_{k}^{\left(\ell \right)}\right)\right\}}_{\ell \in L_{k|k-1}}$, align where the spatial density $p_{k|k-1}^{\left(\ell \right)}\left(x,\tau \right)=\sum_{j=1}^{J_{k|k-1}^{\left(\ell \right)}}\omega_{k|k-1}^{\left(\ell ,j\right)}\delta_{x_{k|k-1}^{\left(\ell ,j\right)},\tau_{k|k-1}^{\left(\ell ,j\right)}}\left(x,\tau \right)$. The updated density is given as $\pi_{k}={\left\{r_{k}^{\left(\ell \right)},p_{k}^{\left(\ell \right)}\left(x_{k}^{\left(\ell \right)},\tau_{k}^{\left(\ell \right)}\right)\right\}}_{\ell \in L_{k}}$, where $p_{k}^{\left(\ell \right)}\left(x,\tau \right)=\sum_{j=1}^{J_{k|k-1}^{\left(\ell \right)}}\omega_{k}^{\left(\ell ,j\right)}\delta_{x_{k}^{\left(\ell ,j\right)},\tau_{k}^{\left(\ell ,j\right)}}\left(x,\tau \right)$ and $L_{k}=L_{k|k-1}$. The normalizing constant $\eta_{z}\left(\ell \right)$ used in Equations (22) and (23) for update can be calculated by:

(37) $$\eta_{z}\left(\ell \right)=\sum_{j=1}^{J_{k|k-1}^{\left(\ell \right)}}\omega_{k|k-1}^{\left(\ell ,j\right)}\gamma_{z}\left(x_{k|k-1}^{\left(\ell ,j\right)}\right)$$

where the $J_{k|k-1}^{\left(\ell \right)}$ covers all particles of track ℓ with different motion models.

Then, the updated existence probability, *i.e*., $r_{k}^{\left(\ell \right)}$, can be obtained as Equation (22), and the weight for each particle, *i.e*., $\omega_{k}^{\left(\ell ,j\right)}$, can be calculated by:

(38) $$\omega_{k}^{\left(\ell ,j\right)}=\frac{\omega_{k|k-1}^{\left(\ell ,j\right)}\gamma_{z}\left(x_{k|k-1}^{\left(\ell ,j\right)}\right)}{\eta_{z}\left(\ell \right)}$$

*Backward Smoothing*: The backward smoothing uses the target density at time l to smooth the particle approximation of the target density and existence probability at time k<l. Suppose the smoothed density from l to k is given as $\pi_{k|l}={\left\{r_{k|l}^{\left(\ell \right)},p_{k|l}^{\left(\ell \right)}\left(x_{k|l}^{\left(\ell \right)},\tau_{k|l}^{\left(\ell \right)}\right)\right\}}_{\ell \in L_{k|l}}$, where $p_{k|l}^{\left(\ell \right)}\left(x,\tau \right)\approx \sum_{j=1}^{J_{k|l}^{\left(\ell \right)}}\omega_{k|l}^{\left(\ell ,j\right)}\delta_{x_{k|l}^{\left(\ell ,j\right)},\tau_{k|l}^{\left(\ell ,j\right)}}\left(x,\tau \right)$. The posterior model transition probability of track ℓ from k−1 to k can be calculated by:

(39) $${\tilde{h}}_{st}^{\left(\ell \right)}=\frac{\sum_{j=1}^{J_{k|l}^{\left(\ell \right)}}\omega_{k|k}^{\left(\ell ,j\right)}\delta \left(x_{k|k}^{\left(\ell ,j\right)},\tau_{k|k}^{\left(\ell ,j\right)}=t,\tau_{k-1|k-1}^{\left(\ell ,j\right)}=s\right)}{\sum_{j=1}^{J_{k|l}^{\left(\ell \right)}}\omega_{k|k}^{\left(\ell ,j\right)}\delta \left(x_{k|k}^{\left(\ell ,j\right)},\tau_{k|k}^{\left(\ell ,j\right)},\tau_{k-1|k-1}^{\left(\ell ,j\right)}=s\right)}$$

The smoothed multi-target LMB density can be calculated by:

(40) $$r_{k-1|l}^{\left(\ell \right)}\approx 1-\left(1-r_{k-1}^{\left(\ell \right)}\right)\times \left(\frac{1-r_{k-1|l}^{\left(\ell \right)}}{1-r_{k|k-1}^{\left(\ell \right)}}\left(1-p_{b}\right)+\frac{r_{k|l}^{\left(\ell \right)}}{r_{k|k-1}^{\left(\ell \right)}}p_{b}\sum_{j=1}^{J_{k|l}^{\left(\ell ,j\right)}}\omega_{k|l}^{\left(\ell ,j\right)}\frac{b_{k|k-1}\left(x_{k|l}^{\left(\ell ,j\right)},\tau_{k|l}^{\left(\ell ,j\right)}\right)}{p_{k|k-1}\left(x_{k|l}^{\left(\ell ,j\right)},\tau_{k|l}^{\left(\ell ,j\right)}\right)}\right)$$

(41) $$p_{k-1|l}^{\left(\ell \right)}\left(x,\tau \right)\approx \sum_{j=1}^{J_{k-1}^{\left(\ell \right)}}{\tilde{\omega }}_{k-1|l}^{\left(\ell ,j\right)}\delta_{x_{k-1}^{\left(\ell ,j\right)},\tau_{k-1}^{\left(\ell ,j\right)}}\left(x,\tau \right)$$

where:

(42) $$\begin{array}{l} {\tilde{\omega }}_{k-1|l}^{\left(\ell ,j\right)}\left(x,\tau \right)\propto \frac{1-r_{k|l}^{\left(\ell \right)}}{1-r_{k|k-1}^{\left(\ell \right)}}\left(1-p_{S,k|k-1}\right)\omega_{k-1}^{\left(\ell ,j\right)}\\ +\frac{r_{k|l}^{\left(\ell \right)}}{r_{k|k-1}^{\left(\ell \right)}}\sum_{n=1}^{J_{k|l}^{\left(\ell \right)}}p_{S,k|k-1}\omega_{k|l}^{\left(\ell ,n\right)}\frac{f_{k|k-1}\left(x_{k|l}^{\left(\ell ,n\right)},\tau_{k|l}^{\left(\ell ,n\right)}|x_{k-1}^{\left(\ell ,j\right)},\tau_{k-1}^{\left(\ell ,j\right)}\right)}{p_{k|k-1}\left(x_{k|l}^{\left(\ell ,n\right)},\tau_{k|l}^{\left(\ell ,n\right)}\right)}\omega_{k-1}^{\left(\ell ,j\right)} \end{array}$$

(43) $$p_{k|k-1}\left(x_{k|l}^{\left(\ell ,n\right)},\tau_{k|l}^{\left(\ell ,n\right)}\right)=\sum_{i=1}^{J_{k-1}^{\left(\ell \right)}}\omega_{k-1}^{\left(\ell ,i\right)}f_{k|k-1}\left(x_{k|l}^{\left(\ell ,n\right)},\tau_{k|l}^{\left(\ell ,n\right)}|x_{k-1}^{\left(\ell ,i\right)},\tau_{k-1}^{\left(\ell ,i\right)}\right)$$

(44) $$f_{k|k-1}\left(x_{k|l}^{\left(\ell ,n\right)},\tau_{k|l}^{\left(\ell ,n\right)}|x_{k-1}^{\left(\ell ,j\right)},\tau_{k-1}^{\left(\ell ,j\right)}\right)={\tilde{h}}_{st}^{\left(\ell \right)}f_{k|k-1}\left(x_{k|l}^{\left(\ell ,n\right)}|x_{k-1}^{\left(\ell ,j\right)},\tau_{k-1}^{\left(\ell ,j\right)}\right)$$

In addition, the resampling, pruning and state extraction are same as the SMC-CBMeMBer [23]. For any two tracks, if the extracted target states are within a given distance threshold $Th_{m}$ the two corresponding LMB components are merged. If the existence probability of ℓ th track is below a threshold $Th_{p}$, the corresponding LMB component is discarded. A target is declared present if the estimated existence probability is greater than threshold $Th_{T}$, otherwise no target is declared. At each time, a maximum of $J_{\max}$ and minimum of $J_{\min}$ particles per-hypothesized track are imposed so that the number of particles representing each hypothesized track is proportional to its existence probability after resampling in the update step.

## 4. Results

### 4.1. Scenario Description

A scenario containing multiple maneuvering targets is used to evaluate the performance of proposed method. The scenario is 30 frames long with period $\Delta_{T}=1$. There are three point targets in this scenario. The point spread function of the simulated sensor has a blurring factor Σ=1 and sensor noise variance is set to σ=1. The image observation consists of 512 × 512 pixels. Each pixel representing one unit of physical distance: $\Delta_{a}=\Delta_{b}=1$. The T(x) is 4×4 square region whose center is closest to the position of x.

**Figure 1:**
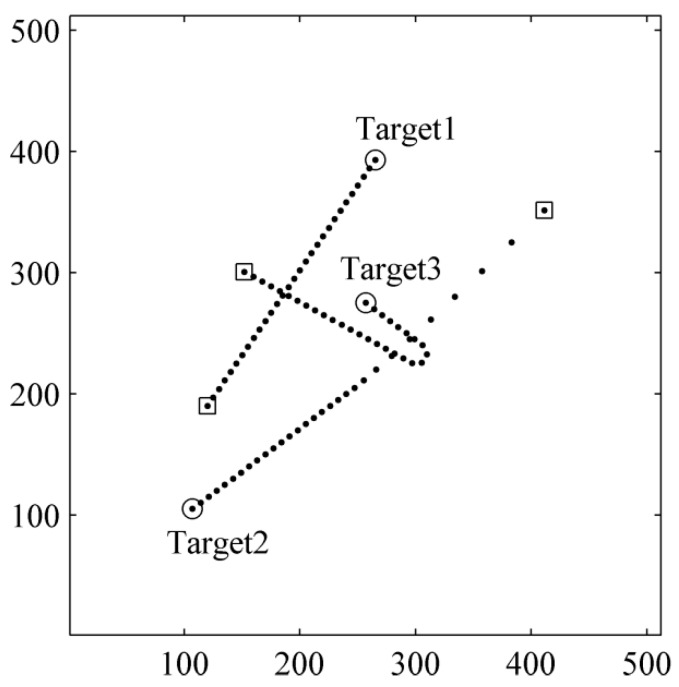
True target trajectories.

**Figure 2:**
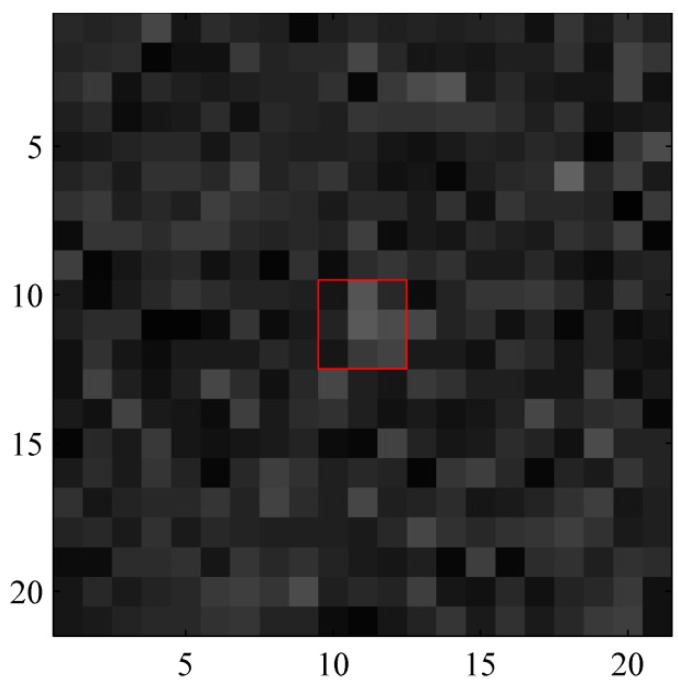
Typical target in image observation at SNR=6.8.

Each of the targets may move at a constant velocity, performing a coordinated turn or under constant acceleration in the surveillance region. Therefore, the motion model set for this example can be composed of a constant velocity (CV) model, a coordinated turn (CT) model and a constant acceleration (CA) model. More details on the motion models can be found in [39]. Let τ=1 denote the CV model, τ=2 denote the CT model and τ=3 denote the CA model. The turn rate of coordinated turn model is set to 1 rad/s, and the acceleration is set to 2.3 pixel/s. The target tracks are shown in Figure 1. “◯” denotes the locations at which targets are born and “□” denotes the locations at which targets die. A typical observation with SNR=6.8 (I=15) is shown in Figure 2. The SNR can be calculated by [34]:

(45) $$SNR=10\log{\left[\frac{I\Delta_{x}\Delta_{y}/2\pi \sum^{2}}{\sigma }\right]}^{2}$$

For the IMM approach, the motion models are initialized with probabilities p(τ=1)=p(τ=2)=p(τ=3)=1/3 and the model transition matrix is set to:

(46) $$H=\left[\begin{array}{ccc} 0.8 & 0.1 & 0.1\\ 0.15 & 0.8 & 0.05\\ 0.15 & 0.05 & 0.8 \end{array}\right]$$

The performance of the algorithm is evaluated using the Optimal Sub-Pattern Assignment (OSPA) metric [40], which is recently developed and defined as:

(47) $${\bar{d}}_{p}^{\left(c\right)}\left(X,Y\right)=\left\{ \begin{array}{ll} 0 & ,m=n=0\\ {\left(\frac{1}{n}\left(\underset{\pi \in \Pi_{n}}{\min}\sum_{i=1}^{m}d^{\left(c\right)}{\left(x_{i},y_{\pi \left(i\right)}\right)}^{p}+c^{p}\left(n-m\right)\right)\right)}^{1/p} & ,m\leq n\\ {\bar{d}}_{p}^{\left(c\right)}\left(Y,X\right) & ,m>n \end{array}\right.$$

where $X=\left\{x_{1},\cdots ,x_{m}\right\}$ and $Y=\left\{y_{1},\cdots ,y_{m}\right\}$ are arbitrary finite subsets, 1≤p≤∞, c>0. We use the parameters c=10 and p=1 in this paper.

The results are divided into two parts: Firstly, multiple maneuvering targets are detected and tracked by TBD-based algorithms, including CV-LMB filter, MM-PHD filter [34,41] and MM-LMB filter. This is used to illustrate the effectiveness of MM-LMB filter for multiple maneuvering targets TBD. Additionally, the performance of MM-LMB TBD with forward-backward combination is compared with the forward MM-LMB filter. It demonstrates the optimization ability of backward smoothing. These TBD methods are implemented using SMC approach. The parameters used in SMC application are listed in Table 1.

**Table 1:** Parameters for SMC TBD.

Variable	$p_{b}$	$p_{S}$	$J_{\max}$	$J_{\min}$	$Th_{m}$	$Th_{p}$	$Th_{T}$
**Value**	0.03	0.99	1200	1000	3	10^−6^	0.5

### 4.2. Multiple Maneuvering Target TBD Experiment

**Figure 3:**
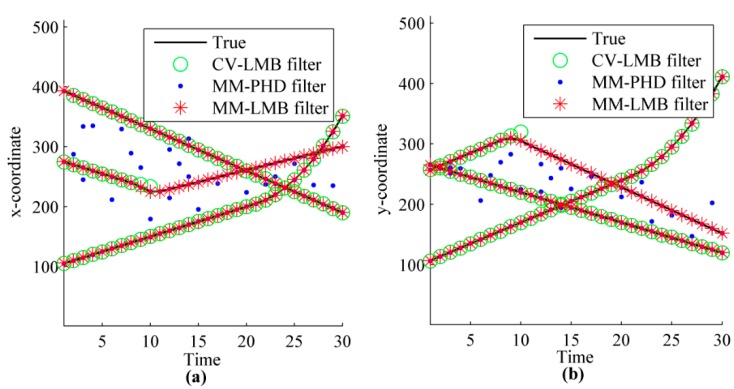
The estimated tracks of multiple targets: (**a**) x-coordinate; (**b**) y-coordinate.

The results obtained from CV-LMB filter, MM-PHD filter and MM-LMB filter for a single run at SNR=6.8 is shown in Figure 3. As indicated by this figure, the tracking performance is quite different. When the motion model switch occurs or the selected model is not consistent with the true model, the CV-LMB filter fails to estimate the targets. The estimation error of MM-PHD filter is also large, because of the inaccuracy in estimating the number of targets. However, the MM-LMB filter can track all targets from birth to end in the above defined scenario.

**Figure 4:**
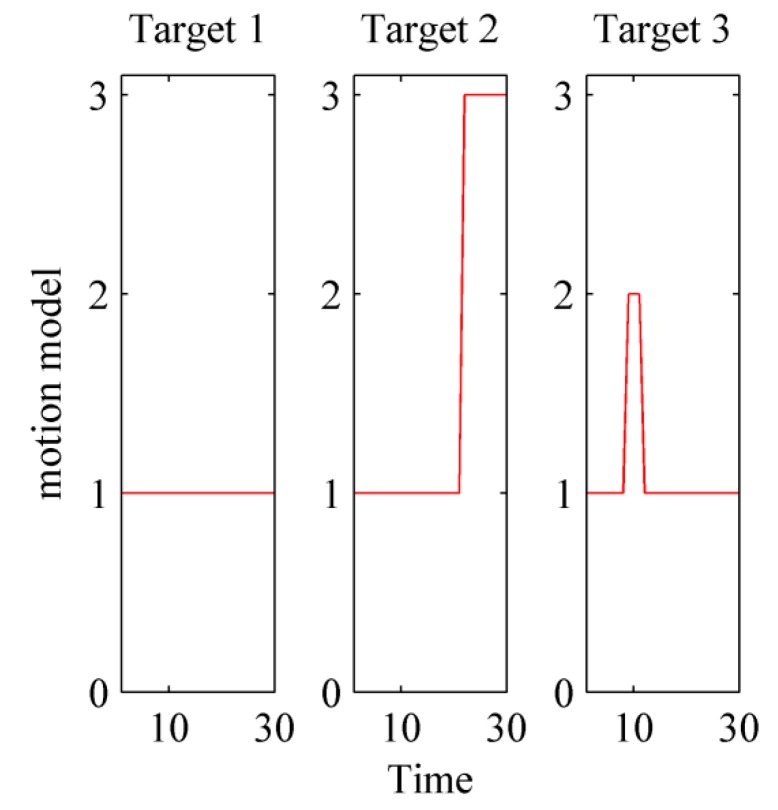
The true motion models of targets.

**Figure 5:**
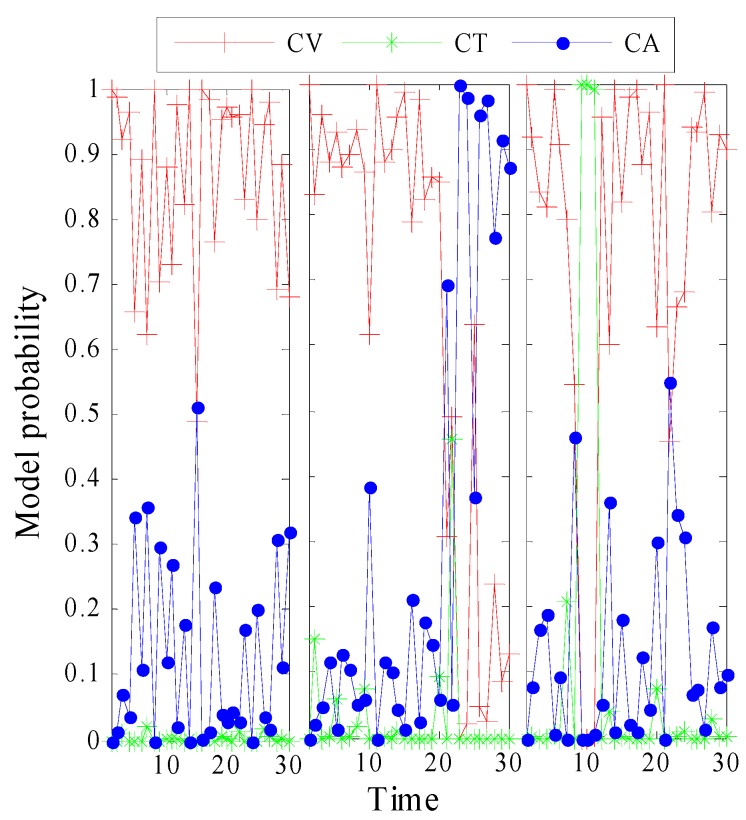
The estimated model probabilities in MM-LMB filter for TBD.

The true motion model and estimated model probability in MM-LMB filter at different times is depicted by Figure 4 and Figure 5, respectively. As expected, the MM-LMB filter can adaptively capture model transition. For example in Target 3, the probabilities of CV, CT and CA at k=8 are 0.53, 0.01, and 0.46, respectively. However, at k=9, the probabilities of CV, CT and CA become 0.005, 0.005 and 0.99. It’s obvious that motion model transition is achieved successfully in MM-LMB filter.

**Figure 6:**
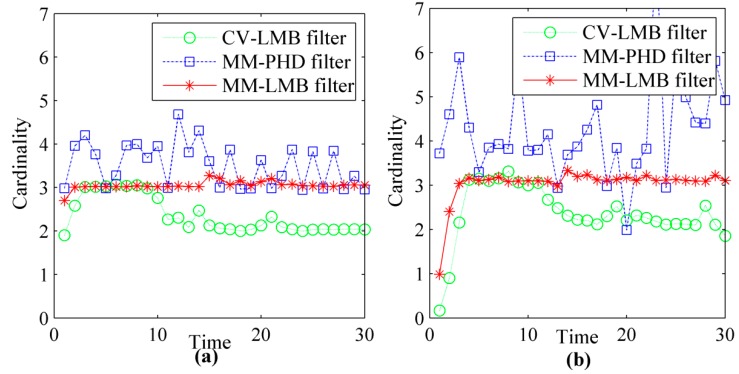
The estimated cardinalities of CV-LMB filter, MM-PHD filter and MM-LMB filter for TBD: (**a**) *SNR* = 6.8; (**b**) *SNR* = 5.

**Figure 7:**
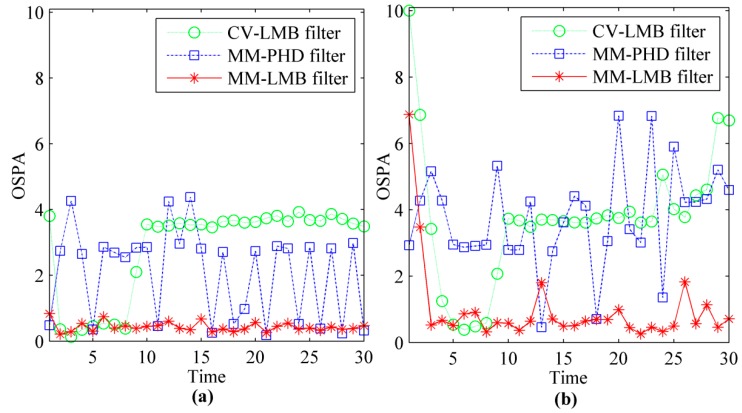
The OSPA distances of CV-LMB filter, MM-PHD filter and MM-LMB filter for TBD: (**a**) *SNR* = 6.8; (**b**) *SNR* = 5.

To provide a performance comparison of the methods in sense of statistical evaluation, the average cardinality and average OPSA distance over 50 Monte Carlo runs at different SNR are shown in Figure 6 and Figure 7. Figure 6a and Figure 7a give the results at SNR=6.8. Figure 6b and Figure 7b give the results at SNR=5, respectively.

From Figure 6, we can see that MM-LMB filter converges to the ground truth. However, the CV-LMB filter and MM-PHD filter produce bias in estimating the target number. MM-LMB filter can effectively capture the model switching property of the maneuvering targets, so its performance is significantly better than that of the other two methods. The average OSPA distances derived by the three methods are shown in Figure 7. From this figure, we can see that CV-LMB filter and MM-PHD filter perform significantly worse than the MM-LMB filter in the term of target localization and detection. In addition, the MM-LMB filter is more effective for dim targets. As shown in Figure 6 and Figure 7, the estimation error may increase as SNR decreases. However, MM-LMB filter still outperforms the other two methods.

### 4.3. Backward Smoothing Experiment

**Figure 8:**
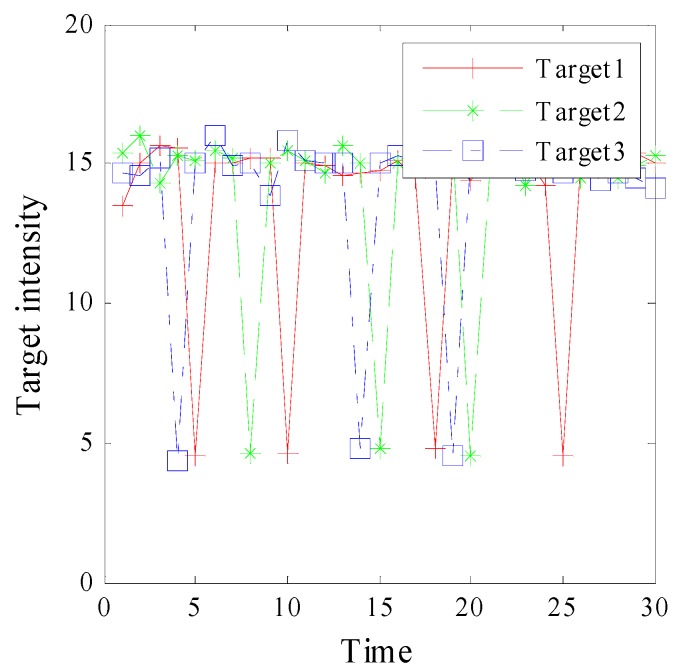
Target intensity variation.

To provide a performance comparison of forward method and forward-backward method, an example simulation is given with target intensity fluctuation. As shown in Figure 8, target intensity degrades significantly at some time, which may lead to significant estimate errors. Besides, 1-lag smoother is adopted in this experiment, it means that the target density at time k is used to smooth the particle approximation of the target density and existence probability at time k−1.

In this experiment, the posterior model transition probabilities at some representative times are listed in Table 2. (s,t) represents the model transition from s to t at time k. $h_{st}$ and ${\tilde{h}}_{st}$ represent the prior and posterior model transition probabilities, respectively. As shown in Table 2, posterior model transition probabilities conform to actual motion models better, especially when motion model switches. For example, Target 3 switches from CV to CT at k=9. The prior transition probability is 0.1, but the posterior one is about 0.8. In addition, Target 3 switches from CT to CV at k=12. The prior transition probability is 0.15, but the posterior one is about 0.85. It proves that the model transition probabilities have been optimized based on posterior target density in smoother.

**Table 2:** Comparison of prior and posterior model transition probabilities.

Target No.	k	(s,t)	$h_{st}$	${\tilde{h}}_{st}$
1	16	(1,1)	0.8	0.98
	26	(1,1)	0.8	0.99
2	11	(1,1)	0.8	0.94
	22	(1,3)	0.1	0.98
3	9	(1,2)	0.1	0.80
	12	(2,1)	0.15	0.85

**Figure 9:**
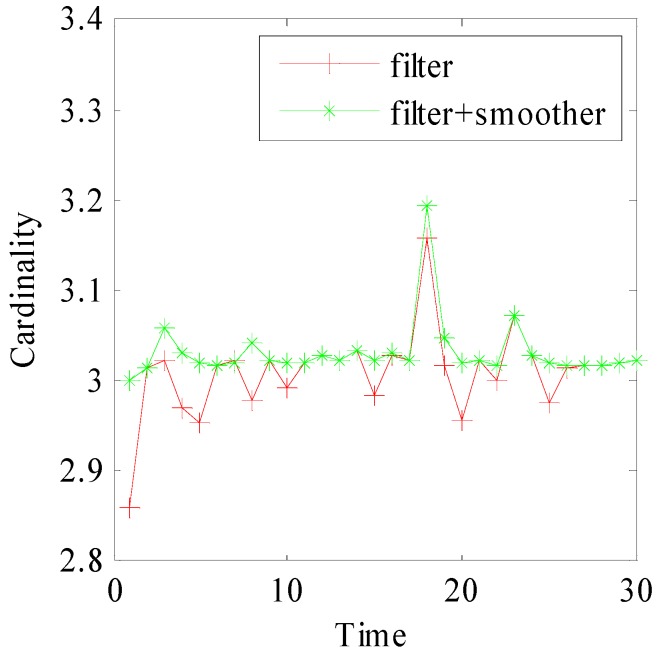
The estimated cardinalities of forward filter and forward-backward combination.

**Figure 10:**
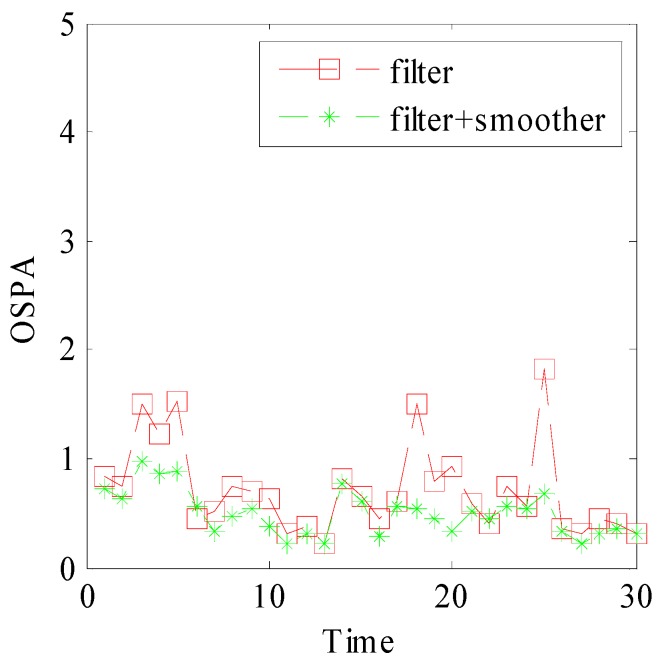
OSPA distances of forward filter and forward-backward combination.

Figure 9 and Figure 10 show the smoothed cardinality and smoothed OSPA distance over 50 Monte Carlo runs. Figure 9 indicates that our method provides accurate cardinality estimation. In Figure 10, the OSPA peaks of filter appear at time k={5,10,15,20,25}, which are related to target intensity fluctuation. However, the result of our method is more stable. This phenomenon indicates that our method is more robust, especially when the target intensity may fluctuate. It’s because that the smoother can exploit current observation to optimize the history result.

## 5. Conclusions

In this paper, we have proposed a labeled RFS-based method, MM-LMB TBD, to detect and track multiple maneuvering targets from raw image observation of infrared focal plane arrays. A forward filter and backward smoother are jointly used to estimate the multi-target states. Simulation results show that the MM-LMB TBD method is capable of tracking maneuvering targets using raw image data. It has better adaptation to maneuvers and provides an overall lower estimate errors compared with the CV-LMB filter and MM-PHD filter. In addition, the smoother of MM-LMB TBD can backwardly optimize the history target state estimation by the current measurement. Thus, MM-LMB TBD is more robust against the target intensity fluctuation problem, which is common in practical applications.
